# Case report: continued treatment with alectinib is possible for patients with lung adenocarcinoma with drug-induced interstitial lung disease

**DOI:** 10.1186/s12890-017-0519-y

**Published:** 2017-12-06

**Authors:** Tatsuya Nitawaki, Yoshihiko Sakata, Kodai Kawamura, Kazuya Ichikado

**Affiliations:** Division of Respiratory Medicine, Saiseikai Kumamoto Hospital, 5-3-1 Chikami, Kumamoto, 861-4193 Japan

**Keywords:** Lung cancer, Non-small cell lung cancer, ALK, Alectinib, Interstitial lung disease, GGO

## Abstract

**Background:**

Alectinib, a second-generation anaplastic lymphoma kinase (ALK) inhibitor, is a key drug for ALK rearranged lung adenocarcinoma. Interstitial lung disease (ILD) is an important adverse effect of alectinib, which generally requires termination of treatment. However, we treated two patients with drug-induced ILD who continued to receive alectinib.

**Case presentation:**

Patient 1 was a 57-year-old male with an *ALK*-rearranged Stage IV lung adenocarcinoma who was administered alectinib as first-line therapy. Computed tomography (CT) detected asymptomatic ground-glass opacity (GGO) on day 33 of treatment. Alectinib therapy was therefore discontinued for 7 days and then restarted. GGO disappeared, and the progression of ILD ceased. Patient 2 was a 64-year-old woman with an *ALK*-positive lung adenocarcinoma who was administered alectinib as third-line therapy. One year later, CT detected GGO; and she had a slight, nonproductive cough. Alectinib therapy was continued in the absence of other symptoms, and GGO slightly diminished after 7 days. Two months later, CT detected increased GGO, and alectinib therapy was continued. GGO diminished again after 7 days. The patient has taken alectinib for more than 2 years without progression of ILD.

**Conclusions:**

Certain patients with alectinib-induced ILD Grade 2 or less may continue alectinib therapy if they are closely managed.

## Background

Alectinib is one of the key drugs for treating patients with ALK-positive non-small cell lung cancer (NSCLC), because it is effective and is well tolerated [1]. Interstitial lung disease (ILD) is an important adverse effect associated with alectinib treatment as well as with all tyrosine kinase inhibitors (TKI) [2]. Generally, when ILD occurs, TKI therapy should be terminated, although treatment with certain molecular targeting inhibitors such as those specific for mammalian target of rapamycin (mTOR) can be continued [3]. It is unclear whether the physician should continue alectinib when ILD is diagnosed. Here we report two patients with alectinib-induced ILD who were able to continue alectinib therapy.

## Case presentation

### Patient 1

**Fig. 1:**
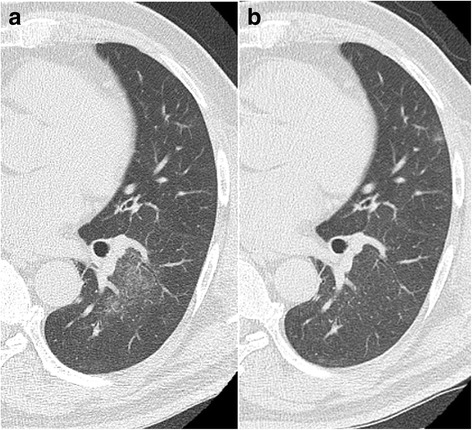
GGO, Case 1. **a** On day 33 of alectinib therapy, a GGO was detected in the left lower lobe. Alectinib was discontinued. **b** On day 61, the GGO disappeared after reintroducing alectinib on day 40

A 57-year-old male smoker with lung adenocarcinoma of his right lower lobe was positive for *EML4-ALK* and positive for ALK. Metastases were present at the third left rib and bilateral adrenal glands, leading to a diagnosis of Stage IV disease. Alectinib (300 mg twice daily) was administered as first-line treatment as per standard of care. CT revealed that the sizes of the primary lesion and other metastases were much smaller compared with baseline. However, 33 days after alectinib administration, CT revealed patchy ground glass opacity (GGO) of the left lower lobe (Fig. 1a). We suspected alectinib-induced ILD, and discontinued treatment. Laboratory data did not detect significant inflammation. Bronchoalveolar lavage fluid (BALF) analysis of the left B9 revealed to be lymphocyte-predominant for 54%. The results of blood tests and microbial culture of the BALF did not indicate any infection (Table 1), so we diagnosed alectinib-induced ILD. But we did not use corticosteroid because he had no symptom of ILD.Alectinib was therefore reintroduced on day 40with careful observation. The GGO in the left lower lobe disappeared on day 61 (Fig. 1b). The patient did not show any symptoms after a 7-month course of alectinib.

**Table 1:** The results of laboratory test of the blood and the bronchoalveolar lavage fluid (BALF)

	Case1	Case2
Blood examination		
WBC (/μL)	6,800	4,500
CRP (mg/dL)	0.02	0.03
LDH (IU/L)	222	202
SP-D (ng/mL)	37	19.3
KL-6 (IU/mL)	299	211
β-D-glucan (pg/mL)	negative	negative
CMV antigen pp65 C7-HRP (antigenemia)	negative	negative
BALF		
Fluid (mL)	17/100	23/150
cell count (/mL)	4 × 10^5^	1 × 10^5^
Lymphocyte (%)	54.5	17
Neutrophil (%)	1	25
Eosinophil (%)	5	1
Basophil (%)	0	0
Histiocyte (%)	39.5	57
CD4/CD8 rate	1.2	0.65
culture	negative	negative
Arterial blood gas		
pH	7.396	NA
PaO_2_ (mmHg)	36.0	NA
PaCO_2_ (mmHg)	82.5	NA
HCO_3_^-^ (mmol/L)	21.6	NA
BE	−2.2	NA

### Patient 2

**Fig. 2:**
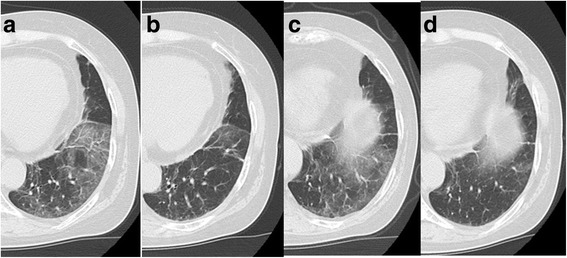
GGO, Case 2. **a** GGO in the left lower lobe 1 year after administration of alectinib. **b** The GGO disappeared after 1 week. **c** Relapse of the GGO after 2 months. **d** The GGO disappeared again after 8 days

A 64-year-old woman nonsmoker had experienced relapsing adenocarcinoma 19 months after undergoing lung surgery and postoperative chemotherapy. She was positive for *EML4-ALK*. Crizotinib therapy was started as first-line treatment. However, multiple lung and brain metastases and carcinomatous lymphagitis developed after 5 months. Whole brain radiotherapy and two cycles of pemetrexed, bevacizumab maintenance therapy after 4 cycles of carboplatin-pemetrexed-bevacizumab therapy were administered. However, 6 months later, brain MRI revealed tumor dissemination to the meninges. Therefore, she received 300-mg alectinib twice daily. Twelve months later, a chest CT revealed GGO in the left lower lobe (Fig. 2a). She presented with a slight, nonproductive cough, no fever or dyspnea, and her blood values were within their normal ranges. Alectinib-induced ILD Grade 2 (Common Terminology Critera for Adverse Events version 4.0) was suspected. Since her symptom was only cough and very slight, we had continued alectinib with careful observation, not using corticosteroid. After 7 days, her cough and the GGO had been improved spontaneously (Fig. 2b), so alectinib treatment was continued. But it became more pronounced 2 months later (Fig. 2c). Then she did not present cough and any other symptoms or abnormal laboratory values. Blood tests and microbial culture of BALF did not detect any infection and cell count of BALF was normal (Table 1). There was no other cause of the GGO, so we diagnosed alectinib-induced ILD. But she was not considered to have significant symptom caused by ILD, we therefore continued alectinib treatment and found that the GGO diminished after 1 week. (Fig. 2d). She has taken alectinib for 2 years without progression of ILD.

## Discussion and conclusions

Alectinib is a key drug for treating patients with *EML4-ALK*-positive NSCLC. In particular, alectinib is highly effective and well tolerated [1], although some patients may experience severe acute ILD [4]. In the ALEX trial and the J-ALEX trial, the incidence of alectinib-induced ILD was reported 1% and 8%, respectively [5, 6]. Physicians generally must discontinue alectinib in such cases, although we believe that certain patients can continue therapy.

In general, drug-induced ILD is suspected when the following criteria are met:(1) history of exposure to the suspected drug, (2) report of suspected previous drug-induced ILD, (3) exclusion of other causes, (4) improvement after discontinuation of a suspected drug, and (5) recurrence of symptoms on rechallenge [7]. Patient 1 did not meet criteria 5 and patient 2 did not meet criteria 4 and 5.; however, there was no evidence of infection and other etiologies of ILD. Furthermore, *Ikeda* et al. reported a patient with alectinib-induced ILD who had GGO and no clinical symptoms, similar to our patients [8]. We conclude therefore that our patients had alectinib-induced ILD.

Generally, drug-induced ILD is characterized by lymphocyte-predominant BALF, while bacterial pneumonia may be associated with neutrophil-predominant BALF. *Ait-Tahar* et al. reported higher immune responses of B and cytotoxic T cells in ALK-positive patients with anaplastic large cell lymphoma than in ALK-negative patients [9]. It is suggested that having ALK fusion gene may lead to higher immune response. Analysis of the BALF of Patient 1 revealed lymphocyte-predominant disease, which might reflect “ALK fusion gene-related drug hypersensitivity”. This “hypersensivity” may lead to the risk of ILD. Otherwise the BALF of Patient 2 was neutrophil-predominant, which may be influenced by the low recovery rate from BALF. Additionally, since there was no evidence of other etiology of the GGO, the absence of lymphocyte-predominant BALF did not exclude drug-induced ILD.

We continued alectinib despite the suspicion of ILD, because patient1 did not have any symptoms, patient 2 had only very sligh cough. *Créquit P* et al. reported that six of 29 patients with ILD who were treated with crizotinib, the first available ALK inhibitor for treating NSCLC, had few clinical symptoms [10]. Moreover, they reported that patients with crizotinib-induced ILD had longer median progression-free survival compared with those without crizotinib-ILD (19.9 vs 6.2 months, *p* = 0.04). Therefore, these findings suggest that a patient with ALK inhibitor-induced ILD may exhibit a higher response compared with those without ALK inhibitor-induced ILD. To our knowledge, this is the first report that patients with ILD Grade 2 or less were able to continue alectinib therapy.

Alectinib may induce severe ILD. For example, CT detected bilateral GGO in a patient with progressive dyspnea [4]. According to *Créquit P* et al., a patient with crizotinib-induced ILD died from acute respiratory distress syndrome on day 28 after the initiation of crizotinib therapy, and this patient previously experienced reversible erlotinib-induced ILD [10]. Therefore, if physicians want to continue alectinib treatment for a patient with alectinib-ILD Grade2 or less, the very careful observation is needed.

There are some limitations to the present study. First, we retrospectively studied two patients who were treated at a single center, and more patients must be studied, or a multicenter prospective study will be required. Second, the recovery rate of BALF was insufficient, so BALF analysis may not have reflected the effect of alectinib for the lungs accurately. Third, it is not clear if our patients responded longer to therapy compared with those without alectinib-induced ILD.

We conclude that **c**ertain patients with alectinib-induced ILD Grade2 or less may continue alectinib therapy if they are closely managed, because they may therefore achieve a prolonged response to therapy that leads to longer survival.

## Abbreviations

ALK: Anaplastic lymphoma kinase
BALF: Bronchoalveolar lavage fluid
CT: Computed tomography
GGO: Ground-glass opacity
ILD: Interstitial lung disease
mTOR: Mammalian target of rapamycin
NSCLC: Non-small cell lung cancer
TKI: Tyrosine kinase inhibitor
